# Clobazam in Adult Drug-Resistant Epilepsy: Excellent Long-Term Retention and Potential Synergy with Neuromodulation

**DOI:** 10.7759/cureus.101154

**Published:** 2026-01-09

**Authors:** Brian Fang, Manar Haroon, Erinn Kalantzis, Mohan Kurukumbi

**Affiliations:** 1 Inova Neuroscience Institute, Inova Health System, Fairfax, USA

**Keywords:** adjunctive therapy, antiepileptic drugs, benzodiazepenes, clobazam, drug-resistant epilepsy, epilepsy, neuromodulation, rational polypharmacy, seizure reduction, vagus nerve stimulation

## Abstract

Introduction: Drug-resistant epilepsy (DRE) is a major clinical challenge. While clobazam, a 1,5-benzodiazepine, is an effective adjunctive therapy, concerns regarding tolerability and discontinuation rates have been reported in some studies. This study evaluates the long-term efficacy and tolerability specifically among adults with DRE who successfully tolerated at least six months of clobazam treatment at a Level 4 Epilepsy Center, providing insights into outcomes in treatment-tolerant patients.

Methodology: A single-center retrospective chart review was conducted of 160 adult patients with DRE treated with clobazam for at least six months at a Level 4 Epilepsy Center. Data on seizure frequency, clobazam dosage, tolerability, and concomitant therapies, including neuromodulation devices, were analyzed.

Results: The cohort (*n* = 160; 80, 50%, female; mean age 37.4 years) achieved significant seizure reduction at a mean clobazam dosage of 20 mg/day. Notably, 62 (38.8%) of patients achieved seizure freedom or near-freedom (≤1 seizure per year). Among patients who tolerated clobazam for at least six months, long-term retention was excellent, with only 5% subsequently discontinuing due to adverse effects. In patients with a vagus nerve stimulator (VNS) or responsive neurostimulation (RNS) device (67, 42% of the cohort), concomitant clobazam therapy resulted in additional reductions in seizure frequency.

Conclusions: Among adults with DRE who successfully tolerate at least six months of clobazam therapy, long-term retention is excellent (95%), and sustained efficacy is maintained. These findings provide valuable insight into long-term outcomes, specifically in treatment-tolerant patients. Furthermore, combination therapy with neuromodulation demonstrates potential complementarity. These findings provide valuable insight into long-term outcomes in treatment-tolerant patients and suggest that careful ongoing management optimizes clinical outcomes in adult DRE.

## Introduction

Epilepsy is a common neurological disorder, but for approximately one-third of patients, seizures persist despite adequate trials of two or more appropriately chosen antiepileptic drugs (AEDs), a condition known as drug-resistant epilepsy (DRE) [1]. The outlook for achieving seizure freedom becomes increasingly modest after multiple failed medication trials, highlighting the urgent need for effective and well-tolerated therapies.

Clobazam, a 1,5-benzodiazepine, possesses a unique pharmacological profile. Unlike traditional 1,4-benzodiazepines, it shows greater affinity for the α2 subunit of the GABA(A) receptor, which is thought to mediate its potent anticonvulsant effects with a more favorable side-effect profile [2]. Furthermore, its primary active metabolite, N-desmethylclobazam, has a long half-life of 71-82 hours, contributing to sustained therapeutic activity [3,4].

Despite its efficacy, questions about clobazam's long-term tolerability have emerged from recent studies. The largest contemporary study on its use in 417 adults with DRE found that while 50.3% of patients had a >50% reduction in seizures, 42.6% ultimately discontinued the medication over the study period [5]. Adverse effects were the primary reason for discontinuation in 55% of cases (approximately 23% of the total cohort). Common side effects included lethargy and fatigue (30.7%) and mood changes (10.8%). Notably, discontinuations occurred predominantly early in treatment, with approximately 30% discontinuing within the first six months. This pattern raises important questions about long-term outcomes among patients who successfully tolerate initial therapy.

This study aimed to evaluate the long-term efficacy and tolerability of clobazam in a cohort of patients who successfully tolerated at least six months of treatment at a single Level 4 Epilepsy Center. We hypothesized that among patients who navigate the initial treatment period, long-term retention would be excellent and sustained efficacy would be maintained.

## Materials and methods

Study design and setting

A single-center retrospective chart review was conducted at Inova's Level 4 Epilepsy Center in Virginia. The medical records of adult patients with a confirmed diagnosis of DRE who were prescribed adjunctive clobazam between 2020 and 2023 were reviewed.

Inclusion and exclusion criteria

Patients were included if they (1) had a confirmed diagnosis of drug-resistant epilepsy, (2) were prescribed clobazam as adjunctive therapy, and (3) remained on clobazam for at least six months. This six-month minimum treatment duration was selected to focus on long-term outcomes in patients who tolerated initial therapy. Patients who discontinued clobazam before six months were not captured in this analysis. 

Patients with incomplete clinical documentation or insufficient follow-up data were excluded from analysis. Missing data handling was by complete-case analysis; patients with missing outcome data at key timepoints were not included in the final cohort of 160 patients.

Data collection

Patients were categorized according to diagnosis, confirmed by long-term electroencephalography (EEG) monitoring and/or evaluation in the epilepsy monitoring unit. Data collection included patient demographics (age and sex), epilepsy diagnosis, number of previously failed AEDs, clobazam starting and current doses, reasons for discontinuation (if applicable), and the presence of adjunctive neuromodulation therapies, including vagus nerve stimulation (VNS) or responsive neurostimulation (RNS), or a history of laser interstitial thermal therapy (LITT).

Outcome measures

The primary outcome measures were (1) changes in seizure frequency as documented in clinical follow-up notes at three- to six-month intervals, (2) the proportion of patients achieving seizure freedom or near-freedom (≤1 seizure per year), and (3) the rate and reasons for treatment discontinuation among the six-month survivor cohort.

Seizure frequencies were categorized as <1 per year, <1 per month, <1 per week, <1 per day, or >1 per day and were compared before and after clobazam initiation. Changes in seizure severity were also documented based on patient and/or caregiver reports.

## Results

Patient characteristics

A total of 160 patients met the inclusion criteria (79, 49.4%, female; 81, 50.6%, male), with a mean age of 37.4 years at the time of data collection (Table 1). The most common diagnosis was focal epilepsy (101, 63%), followed by generalized epilepsy (42, 26%). There were 13 patients (8.1%) with Lennox-Gastaut syndrome and one patient with Dravet syndrome. All patients had failed at least two previous AEDs before clobazam initiation.

**Table 1:** Cohort characteristics. Summary of demographic and clinical variables for the 160 adult patients included in the study. Values are presented as number (percentage) or mean (standard deviation) as indicated. EMU, epilepsy monitoring unit; VNS, vagus nerve stimulation; RNS, responsive neurostimulation; LITT, laser interstitial thermal therapy

Variables	Value
N	160
Age (years), mean (SD)	37.4 (12.5)
Male, *n* (%)	81 (50.4)
Female, *n* (%)	79 (49.4)
EMU stay, *n* (%)	137 (85)
Focal epilepsy, *n* (%)	101 (63)
Generalized epilepsy, *n* (%)	31 (19.4)
Genetic epilepsy, *n* (%)	12 (7.5)
Lennox-Gastaut syndrome, *n* (%)	13 (8.1)
VNS implanted, *n* (%)	62 (38.8)
RNS implanted, *n* (%)	17 (10.6)
LiTT treated, *n* (%)	6 (3.8)

Clobazam dosage

The clinical improvements described later were achieved at a mean maintenance dosage of 10 mg twice daily (20 mg/day), which is consistent with standard therapeutic dosing reported in contemporary adult studies [5]. Review of clinical documentation indicated that most patients maintained stable dosing throughout the follow-up period, with only occasional minor dose adjustments documented. Systematic tracking of dose changes over time was not performed in this retrospective study

Efficacy outcomes

Seizure Freedom and Near-Freedom

A significant proportion of patients (62, 38.8%) achieved excellent seizure control, defined as seizure freedom or near-freedom (≤1 seizure per year).

Seizure Frequency Reduction

**Figure 1:**
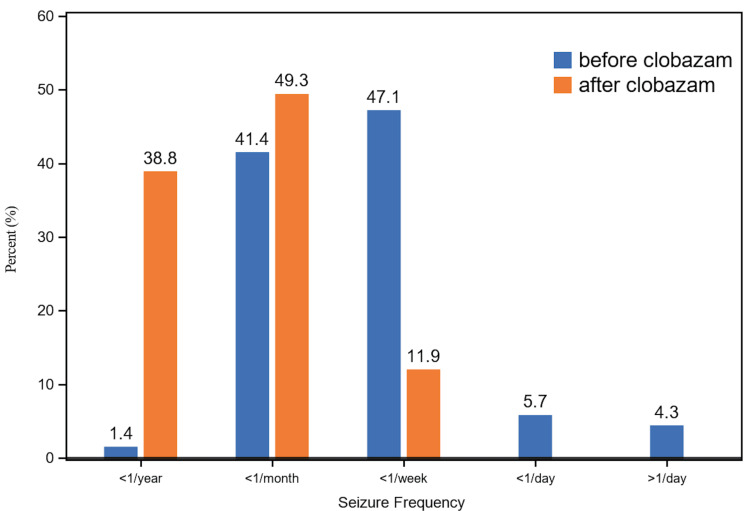
Seizure frequency before and after initiation of clobazam. Seizure frequency at baseline (blue) and at final available follow-up appointment at least one year after clobazam initiation (orange)

The seizure frequency distribution shifted dramatically after clobazam (ONFI) initiation (*P *< 0.001). As shown in Figure 1, the proportion of patients achieving seizure freedom or near-freedom (≤1 seizure per year) increased from 1.4% at baseline to 38.8% (62 patients) after initiation of clobazam. Conversely, patients with high-frequency seizures (daily or multiple daily) experienced complete resolution of this frequency, decreasing to 0% at follow-up.

**Figure 2:**
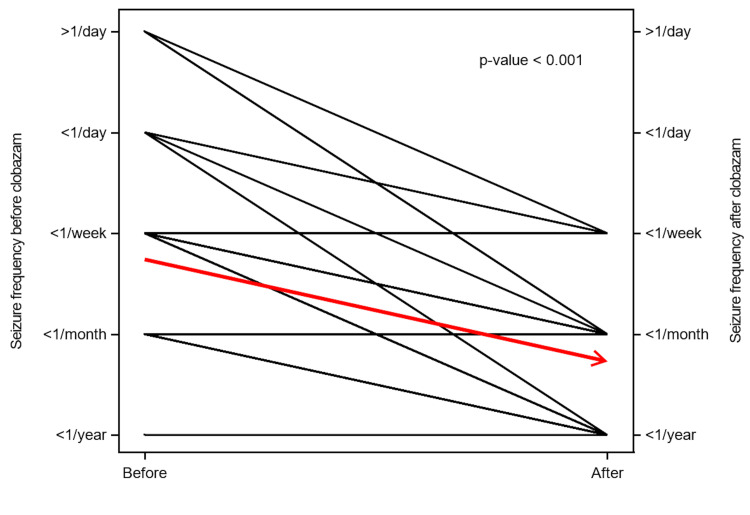
Paired profile for seizure frequency before and after initiation of clobazam.

This reduction was consistent across the cohort. Figure 2 illustrates paired seizure frequency profiles, demonstrating a uniform downward trend in seizure frequency for the vast majority of patients (the red arrow indicates the population mean trend).

Seizure Severity Reduction

Among the 26 (16%) patients who did not achieve a reduction in seizure frequency, 19 (73%) reported a notable decrease in seizure severity (defined as shorter duration, faster recovery, or lighter clustering) based on clinical documentation. This suggests clobazam offers therapeutic value even in patients who do not achieve numeric seizure reduction.

Long-term tolerability and retention

Among the 160 patients who tolerated clobazam for at least six months, 8 patients (5%) discontinued due to adverse effects during extended follow-up; 11 patients (7%) were noncompliant or lost to follow-up; 141 patients (88%) remained on clobazam throughout the study period.

Excluding patients lost to follow-up, the retention rate was 95% (141 of 149 patients with complete follow-up data).

The most commonly reported side effect was mild dizziness, which often resolved without dose adjustment or dissipated after regular use. Other reported side effects were generally mild and manageable.

Potential complementarity with neuromodulation

A substantial portion of the cohort (67, 42%) had an implanted VNS or RNS device (or both) before or during clobazam treatment. In this subgroup, concomitant clobazam therapy resulted in further reduction in seizure frequency. While our retrospective design does not allow for formal quantification of this effect or statistical comparison with patients without devices, this observational pattern suggests potential complementarity between pharmacological and device-based therapies, warranting further investigation.

Outcomes after LITT

Six patients had undergone LITT. Three patients had undergone the LITT procedure due to failure on multiple drugs including clobazam. The other three patients started clobazam after the LITT procedure due to ongoing seizures; all three of these patients achieved seizure freedom after clobazam initiation, demonstrating clobazam's potential value even after advanced surgical interventions. However, the small sample size limits the generalizability of these findings.

## Discussion

This study provides real-world evidence that among adults with drug-resistant epilepsy who successfully tolerate clobazam for at least six months, long-term retention is excellent, sustained efficacy is maintained, and combination with neuromodulation demonstrates promising synergy.

Excellent long-term retention in treatment-tolerant patients

The most significant finding is the excellent long-term tolerability observed in our cohort. Among patients who tolerated clobazam for at least six months, only 8 (5%) subsequently discontinued due to adverse effects during extended follow-up, resulting in an overall retention rate of 95% (141 of 149 patients, excluding patients lost to follow-up) or 88% (141 of 160 patients, including all outcomes).

To contextualize these findings, it is essential to consider the natural history of clobazam discontinuation as described in the literature. The study by Jamil et al. [5] demonstrated that clobazam discontinuations occur predominantly early in treatment: approximately 23% of patients discontinued within the first six months, with 29.8% of all such discontinuations occurring in the first month alone. Among patients who remained on clobazam at six months in that study (approximately 292 patients, or 70% of the original cohort), we estimate that approximately 10% subsequently discontinued due to adverse effects during continued follow-up, though this figure carries substantial uncertainty as it is derived from visual inspection of survival curves rather than explicit reporting.

Understanding selection effects

Our study design, requiring at least six months of clobazam treatment for inclusion, inherently selects for patients who tolerated initial therapy. This is a critical limitation that fundamentally shapes the interpretation of all our findings. Our data provide insights into long-term outcomes among patients who successfully navigate the initial treatment period, rather than outcomes for all patients from treatment initiation. We cannot conclude early tolerability patterns or the proportion of patients who may have discontinued before six months at our center.

Among patients who tolerate initial clobazam therapy (six-month survivors), long-term retention at our center was excellent at 95%. While this compares favorably to the estimated ~10% discontinuation rate among six-month survivors in the Jamil cohort, the uncertainty in that estimate and the many differences between cohorts (patient populations, practice environments, follow-up protocols) preclude definitive conclusions about the reasons for this difference.

Importantly, both our cohort and the Jamil cohort used similar maintenance doses (mean 20 mg/day in our study; median 20 mg/day after initial titration in Jamil, increasing to median 30 mg/day at one year for patients remaining on therapy). This indicates that observed differences in long-term retention are not simply attributable to lower dosing in our cohort.

Sustained efficacy in treatment-tolerant patients

The efficacy observed in our cohort demonstrates that clinically meaningful seizure control can be maintained over extended periods, specifically in treatment-tolerant patients. While our retrospective design does not allow formal assessment of pharmacodynamic tolerance, we observed sustained clinical benefit without obvious loss of effect. The 62 patients (38.8%) who achieved seizure freedom or near-freedom (≤1 seizure per year) represent a substantial clinical benefit in this highly refractory population. A broader shift in seizure frequency distribution was also observed, with 83 (52.1%) patients achieving <1 seizure per year, indicating that meaningful seizure reduction is sustained over time. Additionally, after at least six months of treatment, all patients experienced at least a 50% reduction in seizure frequency, with most achieving substantially greater reductions.

These outcomes are particularly notable given longstanding concerns regarding tolerance development with benzodiazepine therapy. Our findings suggest that among patients who tolerate initial treatment and continue clobazam, clinically meaningful efficacy is maintained without obvious loss of effect during the observation period. While our retrospective chart review methodology cannot exclude the possibility of subtle tolerance that may have been managed through minor dose adjustments or changes in concurrent therapies, we did not observe patterns suggestive of clinically significant tolerance requiring substantial dose escalation or treatment discontinuation. This observation is consistent with published data suggesting that tolerance to clobazam at prescribed therapeutic doses is less pronounced than with traditional 1,4-benzodiazepines, and that clobazam does not meaningfully interfere with the efficacy of rescue benzodiazepines. This observation is consistent with the pharmacological properties of clobazam and its active metabolite, N-desmethylclobazam, which has a long half-life (71-82 hours), allowing for stable plasma concentrations and sustained therapeutic effects [3]. In addition, clobazam’s preferential affinity for α2 GABA(A) receptor subunits over α1 subunits may contribute to sustained efficacy with reduced sedative burden.

Finally, the observation that 19 (73%) patients who did not achieve reductions in seizure frequency nevertheless reported decreased seizure severity underscores that clobazam confers multifaceted clinical benefits beyond frequency reduction alone, with potential meaningful improvements in quality of life through attenuation of seizure intensity.

Mechanistic complementarity in combination therapy

The use of clobazam as adjunctive therapy in this cohort exemplifies the principle of rational polypharmacy in drug-resistant epilepsy. All patients in the cohort were maintained on concurrent antiepileptic medications with complementary mechanisms of action, including SV2A modulators (levetiracetam, brivaracetam), voltage-gated sodium channel blockers (lamotrigine, carbamazepine, oxcarbazepine, lacosamide), and other agents targeting diverse molecular pathways.

Clobazam's unique mechanism, positive allosteric modulation of GABA(A) receptors with preferential α2 subunit binding, provides orthogonal GABAergic enhancement that complements the predominantly glutamatergic or voltage-gated channel mechanisms of most first-line AEDs. This mechanistic diversity is the foundation of rational polypharmacy: combining agents with non-overlapping mechanisms maximizes seizure control while potentially minimizing overlapping toxicities.

The observed efficacy in this cohort, with 38.8% achieving seizure freedom or near-freedom and 52.1% reaching <1 seizure per year, is consistent with the principle of rational polypharmacy: adding GABAergic modulation via clobazam to existing multi-mechanistic regimens may provide complementary seizure control through enhancement of inhibitory neurotransmission. This approach aligns with contemporary epilepsy management strategies that prioritize mechanistically diverse combination therapy over sequential monotherapy trials in refractory populations.

Hypothesis-generating observation: potential complementarity with neuromodulation and post-LITT therapy

A particularly notable observational signal in this cohort is the apparent complementarity between clobazam and neuromodulation devices. While our retrospective design does not permit formal quantification or statistical analysis of interaction effects, clinical documentation suggested additional benefit from combined therapy. Among the 67 (42%) patients with implanted VNS or RNS systems, concomitant clobazam therapy produced additional seizure-frequency reduction, consistent with complementary mechanisms of action: neuromodulation modifies network excitability through focal or distributed stimulation, whereas clobazam enhances global GABAergic inhibition. Together, these modalities may provide more comprehensive suppression of epileptic activity than either approach alone.

This observation has important clinical implications for patients with highly refractory epilepsy and supports consideration of combined pharmacologic and device-based approaches. In addition, three patients achieved seizure freedom after adding clobazam post-LITT, suggesting that clobazam may also contribute meaningfully within broader multimodal treatment strategies, even following advanced surgical interventions. Although this post-LITT effect was not statistically significant due to the very small sample size, it remains a clinically notable signal that warrants further study.

Comparison to published literature

Our long-term retention rate of 95% (excluding lost to follow-up) among six-month survivors compares favorably with other published studies of treatment-tolerant patients. Pediatric studies have reported discontinuation rates of approximately 6%-9% among patients with adequate follow-up [6], and an Indian adult cohort reported 3.6% discontinuation [7], though these studies had shorter follow-up periods. Our findings are consistent with these reports of good long-term tolerability in patients who tolerate initial treatment.

Older studies using somewhat higher mean doses, i.e., 23.9 mg/day [8] and 29.7 mg/day [9], did not provide detailed discontinuation data for direct comparison, although Montenegro et al. [9] reported a one-year retention rate of 61% for all patients from treatment initiation, which likely includes substantial early discontinuation.

Study limitations

This study has several important limitations. The six-month minimum treatment requirement introduces inherent selection bias by excluding patients who discontinued clobazam early; as a result, these findings apply specifically to patients who tolerate initial therapy and cannot be generalized to all patients initiating clobazam. Future prospective studies should include all patients from treatment initiation to characterize both early and late discontinuation patterns. As a single-center retrospective chart review, this study is subject to the limitations of retrospective data collection, including incomplete documentation and the absence of standardized assessment protocols. Seizure frequency was based on patient self-report during clinical visits and is therefore subject to recall bias and reporting inaccuracies; patients may overestimate or underestimate seizure frequency. Similarly, seizure severity assessments relied on subjective patient or caregiver reports, and the use of formal seizure diaries or objective monitoring methods would improve data accuracy. Data on seizure clustering patterns, rescue medication use, and systematic dose titration history were not collected, limiting our ability to assess these potentially important clinical outcomes. Prospective studies should incorporate detailed tracking of these variables. Furthermore, the retrospective design precludes formal assessment of pharmacodynamic tolerance; subtle tolerance requiring minor dose adjustments may not be captured in clinical documentation. Prospective studies with systematic dose tracking and pharmacokinetic monitoring would be needed to definitively characterize tolerance development. 

Findings from a single center with a limited sample size (*n* = 160) may not be generalizable to other practice settings, as institutional protocols, patient populations, practice patterns, and demographic characteristics may differ. Multi-center studies with larger, more diverse cohorts are needed to validate these findings across different populations and geographic regions. Future studies should include comprehensive demographic collection to evaluate potential population-specific differences in clobazam efficacy and safety.

In the absence of a control group, it is not possible to definitively attribute observed outcomes to clobazam therapy or to distinguish treatment effects from patient characteristics or other confounding factors. The estimated ~10% discontinuation rate among six-month survivors in the Jamil cohort is subject to substantial uncertainty, as it was derived from visual inspection of survival curves rather than explicitly reported data, which limits the strength of cross-study comparisons. Finally, detailed demographic data on race/ethnicity were not systematically collected, limiting our ability to assess whether treatment response or tolerability varies across different ethnic populations. 

Clinical implications

For clinicians managing patients with drug-resistant epilepsy, these findings suggest that patients who successfully tolerate clobazam during the initial six-month period have excellent prospects for long-term retention with sustained efficacy. Clobazam may be considered in patients with neuromodulation devices based on the hypothesis-generating observation that concomitant therapy may provide additional benefit, though formal prospective studies are needed to confirm this potential interaction. Careful attention to dose titration, patient education, and proactive management of emerging side effects may optimize long-term outcomes. Clobazam remains an important adjunctive therapy and may complement LITT to further improve seizure outcomes. Quality-of-life considerations are paramount, as the ideal outcome combines seizure freedom with minimal adverse effects.

Advocacy for expanded FDA approval

Currently, clobazam is FDA-approved only for Lennox-Gastaut syndrome. Our findings, in combination with accumulating evidence from other centers (including larger multi-center studies), suggest efficacy across a broader range of epilepsy types, including focal epilepsy, which comprised 101 (63%) of our cohort, and generalized epilepsy. While single-center retrospective studies such as ours are insufficient for regulatory decision-making, the growing body of real-world evidence may eventually support consideration of expanded FDA approval, which would improve insurance coverage and accessibility for patients who may benefit from clobazam as adjunctive therapy. However, such regulatory expansion would appropriately require prospective, multi-center trials with rigorous methodology.

Future directions

Future research should address the limitations of this study through:

(1) Prospective, multi-center studies: Capturing all patients from treatment initiation across multiple centers with diverse patient populations and geographic regions to better characterize early discontinuation patterns, validate our findings regarding long-term retention in treatment-tolerant patients, and identify predictors of successful long-term treatment

(2) Comparative effectiveness studies: Examining whether specific titration protocols, patient education strategies, or monitoring approaches improve both early and late retention rates

(3) Mechanistic studies: Investigating the mechanisms underlying apparent synergy between clobazam and neuromodulation devices

(4) Long-term follow-up studies: Evaluating efficacy and tolerability over periods exceeding two years to assess very long-term outcomes

(5) Standardization of seizure reporting: Implementing standardized seizure frequency and severity assessment tools to improve accuracy of outcome measures (6) Assessment of additional clinical outcomes: Evaluating the impact of clobazam on seizure clustering patterns and rescue medication use, which may provide additional insights into quality-of-life benefits beyond seizure frequency reduction. These outcomes warrant investigation in future prospective studies or as the subject of a separate retrospective analysis.

## Conclusions

This study demonstrates that specifically among adults with drug-resistant epilepsy who successfully tolerate at least six months of clobazam treatment, long-term retention is excellent, with only 5% subsequently discontinuing due to adverse effects. Sustained efficacy is maintained over extended follow-up, with 62 (38.8%) patients achieving seizure freedom or near-freedom (≤1 seizure per year) and 79 (49.3%) experiencing fewer than one seizure per month. The observed pattern suggesting potential complementarity between clobazam and neuromodulation devices represents a hypothesis-generating finding that warrants formal investigation through prospective controlled studies.

While the study design inherently selects for patients who tolerate initial therapy and therefore cannot address early discontinuation patterns or provide guidance on overall outcomes from treatment initiation, these results provide valuable hypothesis-generating insights into long-term outcomes specifically among treatment-tolerant patients. Clobazam at standard therapeutic doses (approximately 20 mg/day) should be considered a valuable long-term treatment option for adults with drug-resistant epilepsy, particularly when used in combination with neuromodulation therapies.

Future prospective, multi-center studies that include all patients from treatment initiation are needed to validate these promising findings in diverse populations, fully characterize the efficacy-tolerability profile of clobazam across all phases of treatment, and identify strategies that optimize both early tolerance and long-term retention. Such validation studies would support evidence-based incorporation of clobazam into formal treatment protocols for drug-resistant epilepsy.
